# COVID-19-Induced Encephalopathy: A Case Report

**DOI:** 10.7759/cureus.41788

**Published:** 2023-07-12

**Authors:** Kunal Karmilkar, Aditi Patel, Nehal Patel

**Affiliations:** 1 Neurology, Edward Via College of Osteopathic Medicine, Monroe, USA; 2 Internal Medicine, Rapides Regional Medical Center, Alexandria, USA

**Keywords:** covid induced encephalopathy, neurology and critical care, atypical presentation, covid-19 and seizure, covid-19

## Abstract

While COVID-19 is known to cause common neurological manifestations such as loss of taste and smell, headaches, and myalgias, rare and severe neurological complications can also occur. We describe the hospitalization of a middle-aged Caucasian woman who presented with altered mental status and an absence of moderate-severe pulmonary symptoms. The patient tested positive for COVID-19 and experienced a tonic-clonic seizure six days after admission. Diagnostic testing, including cerebrospinal fluid analysis, blood cultures, urine cultures, brain imaging, and electroencephalograms were unremarkable, indicating a global encephalopathic state. This case highlights the need for clinicians to anticipate neurological complications when managing patients with COVID-19, especially when respiratory symptoms are minimal or absent. Moreover, further research on COVID-19-induced encephalopathy is crucial to improve patient outcomes and inform clinical practice.

## Introduction

Near the end of 2019, a novel Coronavirus was discovered in Wuhan, China. Suddenly cases of pneumonia accumulated in this region and the disease promptly spread across the globe. By February of 2020, the World Health Organization (WHO) identified this strain as COVID-19 [1]. As of March 2023, in the United States, there have been over 100 million total cases and over 1.1 million total deaths reported of COVID-19 [2]. While global efforts proceed to combat this disease, it continues to evolve along with our comprehension of its clinical course. One particular complication that is increasingly being recognized with COVID-19 is its neurological manifestations. Even in the absence of typical respiratory symptoms, 36.4% of patients have been observed to have some form of neurological illness [3,4]. Frequent neurological complications include anosmia, ageusia, headaches, myalgia, and myositis. Infrequent neurological complications reported include encephalopathy, encephalitis, cerebrovascular disease, Guillain-Barre syndrome, and hemophagocytic lymphohistiocytosis [3,4]. Physicians should foresee neurological compilations when managing patients with COVID-19. Here we present a case of COVID-19-induced encephalopathy with new-onset generalized tonic-clonic seizures and complex partial seizures.

## Case presentation

A 60-year-old Caucasian female presented to the emergency room unconscious and unresponsive to stimuli. Prior to arrival, she was administered Narcan with no change in status. Upon arrival at the hospital, she tested positive for COVID-19 and had a blood sugar of 122. She has a significant past medical history of hypertension, diabetes mellitus, gastroesophageal reflux disease, cerebrovascular accident, neuropathy, and opiate addiction. On presentation, the patient was afebrile, heart rate of 127 beats/minute, blood pressure of 160/112 mm Hg, respirations of 12 breaths/minute, and oxygen saturation of 93% on room air. Neurologic examination revealed an obtunded state, fixed rightward gaze, subtle movement of the right lower extremity, and minimal withdrawal response to stimuli. All other physical exam findings were within normal limits. Concerned for meningitis/encephalitis, the patient was also started on empiric broad-spectrum antibiotics. During the first few days of the hospital course, there were concerns for underlying complex partial seizures. Antiepileptic agent (AED), intravenous (IV) levetiracetam was initiated and IV midazolam added as needed for seizures. Concern for complex partial seizures remained given the patient's presentation. Six days post-admission, the patient experienced a tonic-clonic seizure that was self-aborted but symptoms of a postictal state shortly followed.

Investigations

On admission, a complete metabolic panel (CMP) demonstrated a sodium of 138, potassium of 4.8, chloride of 100, bicarbonate of 18, blood urea nitrogen of 30, creatinine of 1.64, and glucose of 197. Complete blood count with differential (CBC w DIFF) showed a normocytic, normochromic picture and a white blood count of 10.2. Her alcohol, ammonia, urinalysis, arterial blood gas, thyroid stimulating hormone, lipid profile, and folate levels were noncontributory. Lactic acid was 5.1, creatinine kinase was 708, troponin T was <0.01, creatine kinase-MB was 13.7, myoglobin was 768.5, vitamin B12 was 939, serum osmolality was 307, and procalcitonin was 2.81. Urine toxicology screen came back negative. On day 3 of admission, the patient underwent lumbar puncture (LP) for further cerebrospinal fluid (CSF) studies, which was clear with mildly elevated glucose (87), 0 WBCs, normal total protein levels, and unremarkable cultures or other CSF studies. On the day of her seizure, her labs were redrawn and her CMP was within normal limits except for her glucose which was 145 and glomerular filtration rate of 63. Her magnesium and phosphorus levels were within normal limits as well. CBC w DIFF now showed a macrocytic, normochromic picture with an uptrending white blood cell count of 13.8. Renal panel, blood culture, and urinalysis were unremarkable.

**Figure 1:**
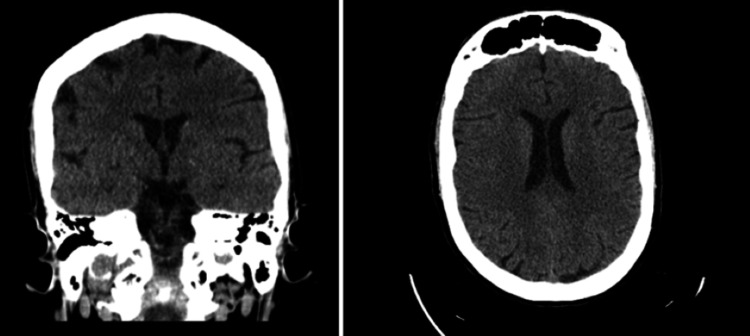
CT head without contrast showed no significant abnormality.

**Figure 2:**
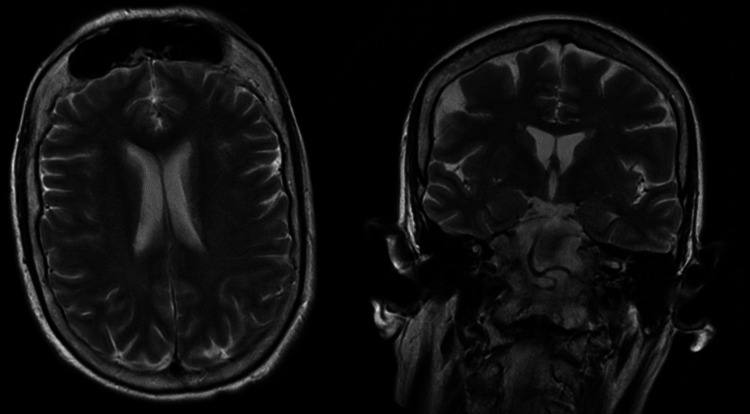
MRI of the head was unremarkable.

**Figure 3:**
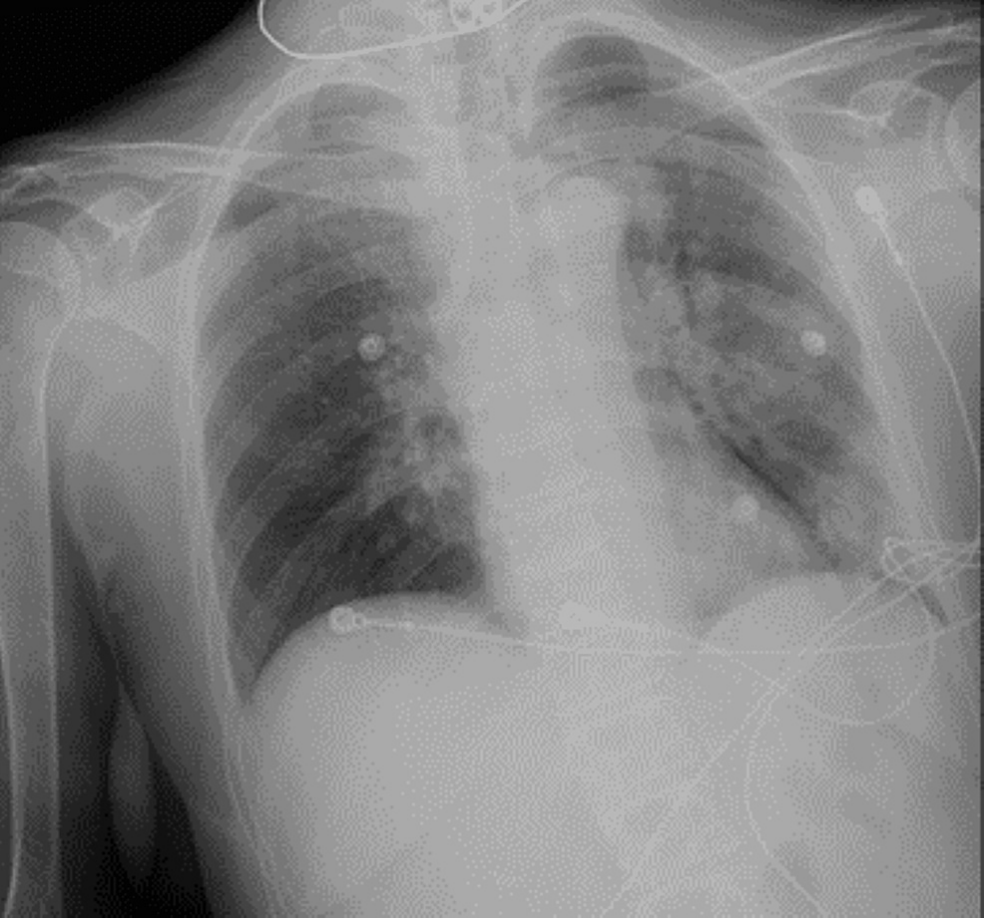
CXR showing left lower lobe pneumonia. CXR: Chest X-ray

**Figure 4:**
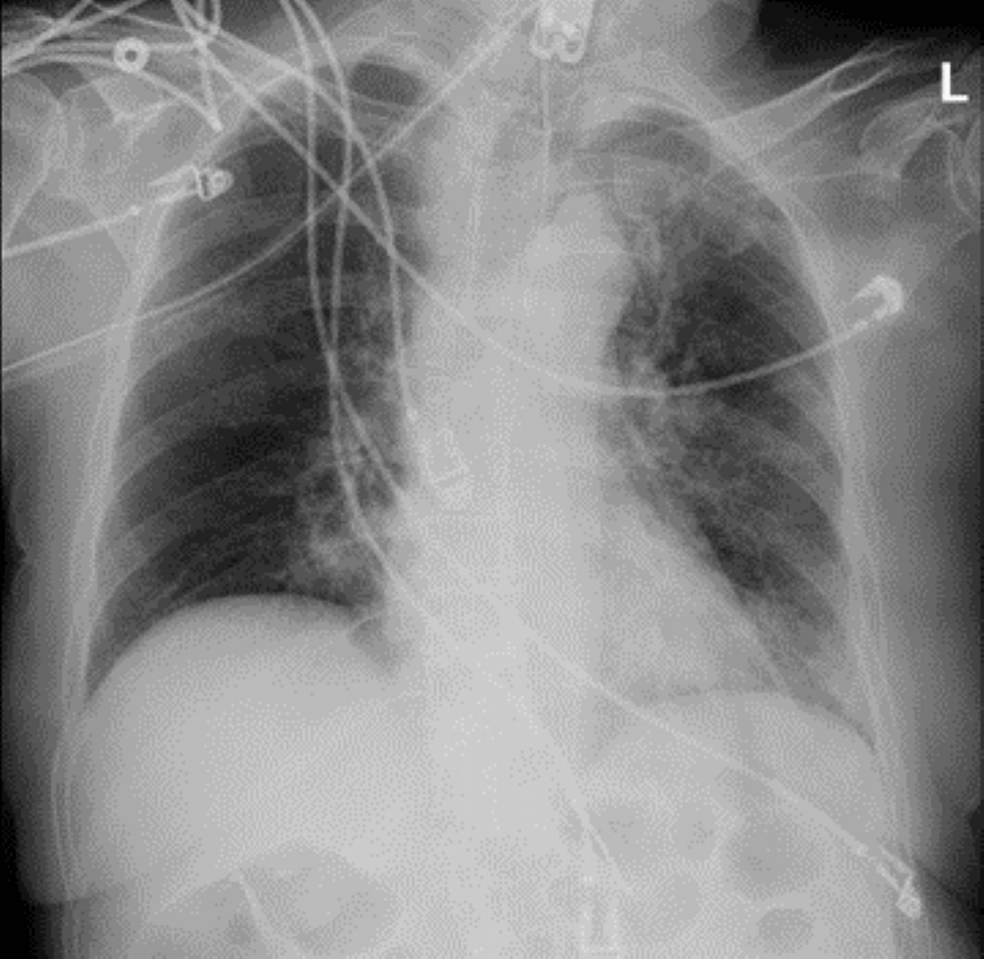
CXR showing improvement of left lower lobe pneumonia. CXR: Chest X-ray

**Figure 5:**
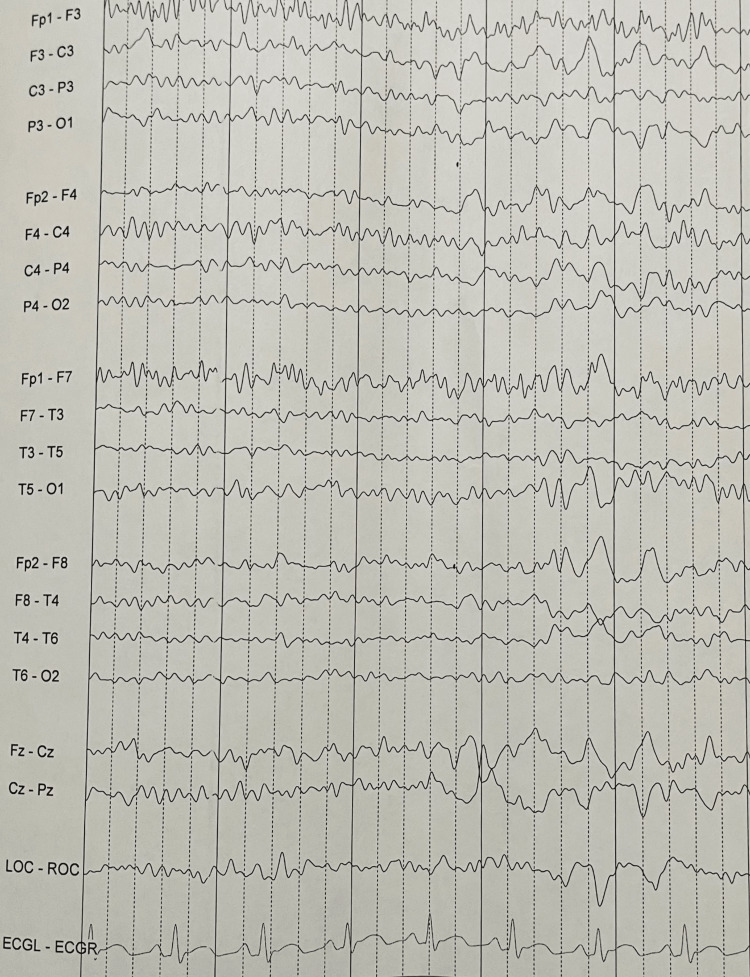
Abnormal EEG compatible with encephalopathy of unknown etiology.

**Figure 6:**
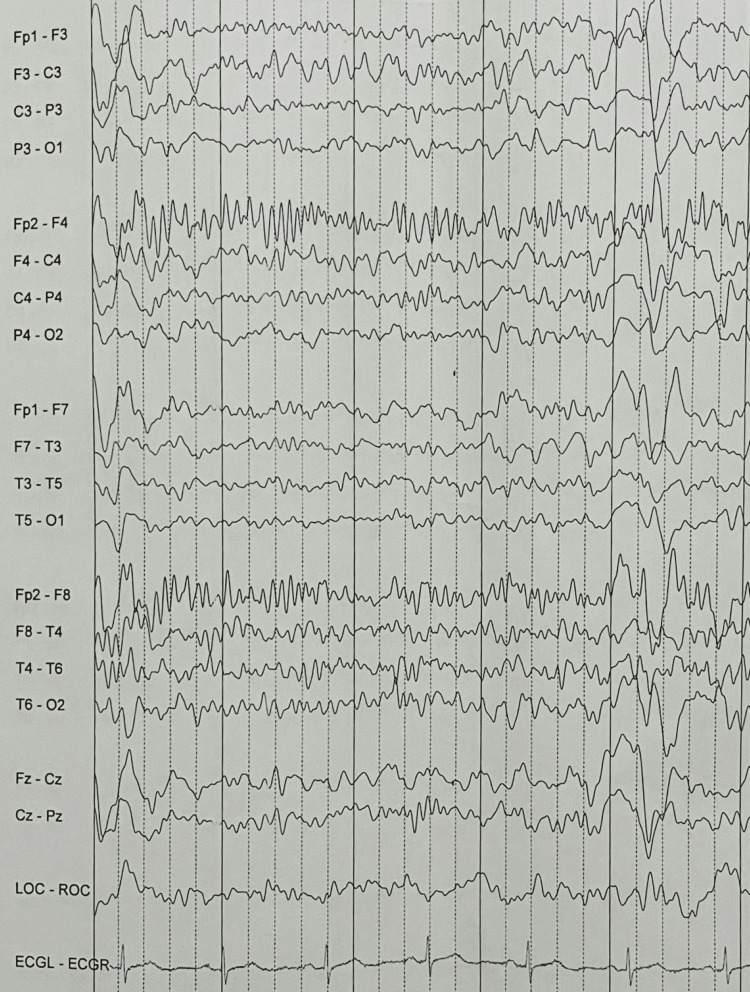
Abnormal EEG compatible with encephalopathy of unknown etiology or post-ictal state.

CT head without contrast on admission demonstrated that ventricles were of normal size. No hemorrhage, edema, or extra-axial blood collections were seen. The brainstem, cerebellum, sinuses, mastoids, and calvarium were normal (see Figure 1). MRI of the brain without contrast showed no evidence of acute ischemia or infarct. There was no evidence of mass lesion, abnormal intra or extra-axial fluid collection, acute hemorrhage, or other acute process (see Figure 2). Initial chest X-ray (CXR) showed expanded lungs with patchy diffuse left lower lobe infiltrates indicative of pneumonia. Heart size was normal and bones were age-appropriate (see Figure 3). A repeat chest X-ray three days later showed improved aeration into the left lower lung with no other abnormalities (see Figure 4). An initial EEG of 1-hour duration showed an abnormal result compatible with encephalopathy of unknown etiology or postictal state (see Figure 5). The patient underwent another EEG prior to her seizure which was noted to be unremarkable other than signs of global encephalopathy (see Figure 6).

## Discussion

There has been an increasing number of reports in the medical community concerning the occurrence of seizures in patients with COVID-19 of which the first was by Moriguchi et al. [5]. The mechanism of seizures in association to COVID-19 infection is still not fully understood. Some studies propose viral entry through the olfactory nerve via retrograde transport or indirect mechanisms that may lead to focal cerebral inflammation [6,7]. In a study, Nikbakht et al. suggested that neurological symptoms in COVID-19 mainly arise in response to pro-inflammatory cytokine destruction of the central nervous system (CNS). These cytokines are responsible for disrupting the blood-brain barrier, impairing ion channel function, and causing an imbalance of excitatory and inhibitory neurotransmitters. As a result, the cytokines have been shown to cause lingering CNS damage post viral infection [8,9]. The residual inflammation caused by the virus may explain the new onset seizures observed in our patient with no other identifiable etiology.

Appropriate diagnostic tools were utilized to work up common causes of seizures before considering COVID-19 encephalopathy as a cause of new-onset seizures in our patient. Initially, we suspected a possible drug overdose. The patient was administered Narcan with no change in mental status. There have been many studies suggesting that electrolyte abnormalities in COVID-19 patients may lead to seizures, specifically hyponatremia, hypomagnesemia, and hypocalcemia [10,11]. However, our patient displayed no electrolyte abnormalities, and her labs were within normal limits. Another probable cause considered was sepsis-induced encephalopathy which can lead to seizures. Alessandri et al. suggested that the release of a cytokine storm leads to increased excitatory neurotransmitters in the nervous system which may make a septic patient more susceptible to seizures [12]. Upon admission, our patient’s chest X-ray showed a left lower lobe pneumonia, however, she was initially treated with broad-spectrum antibiotics ceftriaxone, metronidazole, and azithromycin and then switched over to vancomycin, cefepime, and trimethoprim-sulfamethoxazole. A three-day follow-up chest X-ray showed a clearing of the left lower lung with no other abnormalities. Our patient's lumbar puncture, blood culture, and urinalysis did not grow any organisms. Moreover, our patient did not meet the SIRS or qSOFA sepsis criteria and severe sepsis screening in the hospital was negative.

An additional studied cause of seizures includes post-stroke epilepsy [13]. Our patient suffered from a cerebral vascular accident two months prior however she had no history of epilepsy or seizures. Moreover, her CT head without contrast showed no acute processes, and MRI of the brain without contrast was unremarkable. With no prior history of seizures, we ruled out a cerebral insult as the cause of our patient's seizures. Multiple EEG studies were done on our patient and all failed to show epileptiform activity. However, the EEG studies were abnormal in that they suggested encephalopathy of unknown etiology or postictal state. Clinical improvement on AED, along with results from our EEG studies and imaging, supported our diagnosis that our patient was suffering from new onset seizures. The patient was administered levetiracetam and midazolam as needed for seizures and observed in the hospital for a few more days. The patient showed signs of neurologic recovery and experienced no additional seizure activity after day 6. Unremarkable labs, negative urine toxicology screening, and negative sepsis screening in our patient ruled out other common causes of seizures. We suspect that this patient’s new onset seizure may have been a result of residual inflammatory insult to the CNS from her COVID-19 infection.

## Conclusions

In summary, this case report serves as a crucial reminder of the intricate relationship between COVID-19 and the central nervous system. The presented neurological complications underscore the necessity of vigilance among healthcare professionals in recognizing and promptly treating non-respiratory manifestations of COVID-19. The hypothesis that pro-inflammatory cytokine destruction may have contributed to the observed seizures highlights the complexity of the disease's pathogenesis, which requires further investigation. Ultimately, the findings of this report highlight the urgent need for continued research on COVID-19-induced encephalopathy to inform clinical practice and improve patient outcomes.
